# Effect of Glyceryl Trinitrate on Hemodynamics in Acute Stroke: Data From the ENOS Trial

**DOI:** 10.1161/STROKEAHA.118.023190

**Published:** 2019-01-10

**Authors:** Jason P. Appleton, Lisa J. Woodhouse, Daniel Bereczki, Eivind Berge, Hanne K. Christensen, Rónán Collins, John Gommans, George Ntaios, Serefnur Ozturk, Szabolcs Szatmari, Joanna M. Wardlaw, Nikola Sprigg, Peter M. Rothwell, Philip M. Bath

**Affiliations:** 1 From the Stroke Trials Unit, Division of Clinical Neuroscience, University of Nottingham, United Kingdom (J.P.A., L.J.W., N.S., P.M.B.); 2 Department of Stroke, Nottingham University Hospitals NHS Trust, United Kingdom (J.P.A., N.S., P.M.B.); 3 Department of Neurology, Semmelweis University, Budapest, Hungary (D.B.); 4 Department of Internal Medicine and Cardiology, Oslo University Hospital, Norway (E.B.); 5 Department of Neurology, Bispebjerg and Frederiksberg Hospital, Copenhagen, Denmark (H.K.C.); 6 Stroke Services, Trinity College Dublin, Tallaght Hospital, Ireland (R.C.); 7 Department of Medicine, Hawke’s Bay District Health Board, Hastings, New Zealand (J.G.); 8 Department of Medicine, University of Thessaly, Larissa, Greece (G.N.); 9 Department of Neurology, Selcuk University Faculty of Medicine, Konya, Turkey (S.O.); 10 Department of Neurology, Clinical County Emergency Hospital, Targu Mures, Romania (S.S.); 11 Division of Neuroimaging Sciences, Centre for Clinical Brain Sciences, UK Dementia Research Institute at the University of Edinburgh, (J.M.W.); 12 Nuffield Department of Clinical Neurosciences, John Radcliffe Hospital, University of Oxford, United Kingdom (P.M.R.).

**Keywords:** blood pressure, glyceryl trinitrate, heart rate, hemodynamics, hemorrhage

## Abstract

**Background and Purpose—:**

Increased blood pressure (BP), heart rate, and their derivatives (variability, pulse pressure, rate-pressure product) are associated with poor clinical outcome in acute stroke. We assessed the effects of glyceryl trinitrate (GTN) on hemodynamic parameters and these on outcome in participants in the ENOS trial (Efficacy of Nitric Oxide in Stroke).

**Methods—:**

Four thousand and eleven patients with acute stroke and raised BP were randomized within 48 hours of onset to transdermal GTN or no GTN for 7 days. Peripheral hemodynamics were measured at baseline (3 measures) and daily (2 measures) during treatment. Between-visit BP variability over days 1 to 7 (as SD) was assessed in quintiles. Functional outcome was assessed as modified Rankin Scale and cognition as telephone mini-mental state examination at day 90. Analyses were adjusted for baseline prognostic variables. Data are mean difference or odds ratios with 95% CI.

**Results—:**

Increased baseline BP (diastolic, variability), heart rate, and rate-pressure product were each associated with unfavorable functional outcome at day 90. Increased between-visit systolic BP variability was associated with an unfavourable shift in modified Rankin Scale (highest quintile adjusted odds ratio, 1.65; 95% CI, 1.37–1.99), worse cognitive scores (telephone mini-mental state examination: highest quintile adjusted mean difference, −2.03; 95% CI, −2.84 to −1.22), and increased odds of death at day 90 (highest quintile adjusted odds ratio, 1.57; 95% CI, 1.12–2.19). GTN lowered BP and rate-pressure product and increased heart rate at day 1 and reduced between-visit systolic BP variability.

**Conclusions—:**

Increased between-visit BP variability was associated with poor functional and cognitive outcomes and increased death 90 days after acute stroke. In addition to lowering BP and rate-pressure product, GTN reduced between-visit systolic BP variability. Agents that lower BP variability in acute stroke require further study.

## Abstract 2

Supplemental Digital Content is available in the text.

Elevated blood pressure (BP) is present in 75% of patients with acute stroke^1^ and is associated with increased death and poor functional outcome in all stroke types,^2,3^ recurrent stroke in ischemic stroke,^4^ and hematoma expansion in intracerebral hemorrhage (ICH).^5^ Increased heart rate (HR) is similarly associated with poor outcome after acute stroke.^6^ Mathematical derivations of BP and HR provide useful summaries of hemodynamic parameters and include peak systolic BP (SBP), mean arterial pressure (MAP), pulse pressure (PP), PP index (PPI), rate-pressure product (RPP), and variability in each of them. Each parameter is associated independently with a worse functional outcome, death, recurrent stroke, or early neurological deterioration.^7–10^ Variability may be assessed across a set of measurements taken at 1 visit (within-visit) or across several visits (between-visit), with different antihypertensive drug classes having variable effects on BP variability in the outpatient setting.^11,12^

Recent large trials assessing whether BP should be lowered in acute ischemic stroke were neutral.^13–15^ In contrast, lowering BP in ICH was associated with improved functional outcome in the INTERACT-2 (Intensive Blood Pressure Reduction in Acute Cerebral Hemorrhage Trial-2)^16^ but had a neutral effect in the ATACH-2 trial (Antihypertensive Treatment of Acute Cerebral Hemorrhage-2).^17^ Small phase II trials of glyceryl trinitrate (GTN, a nitric oxide donor) in acute or subacute stroke found that it lowered peripheral and central BP, 24 hour BP, peak SBP, PP and PPI; increased HR; improved vascular compliance; and did not change cerebral blood flow or velocity or increase intracranial pressure.^18–21^ Although GTN did not modify outcome overall in the large ENOS trial (Efficacy of Nitric Oxide in Stroke),^15^ patients randomized to GTN within 6 hours of onset showed a significant improvement in functional outcome.^22,23^

We assessed the association between hemodynamic measures and outcome and the hemodynamic effects of GTN in acute stroke using data from the ENOS trial.^15^

## Methods

Details on the ENOS trial protocol, statistical analysis plan, baseline characteristics, and main trial results have been published elsewhere.^15,24–26^ In brief, ENOS recruited 4011 patients within 48 hours of onset of stroke symptoms with high SBP (140–220 mm Hg) to transdermal GTN (5 mg patch) or no patch for 7 days. In addition, participants taking antihypertensive medication before their stroke were randomized to continue or stop these drugs for 7 days. Key exclusion criteria included definite need to start, continue, or stop, BP-lowering medications; need for, or contraindication to, GTN; Glasgow Coma Scale <8; pure sensory stroke; isolated dysphasia; preceding moderate or severe dependency (modified Rankin Scale [mRS] 3–5); or a condition mimicking stroke.^15^ Patients or relatives/carers gave written informed consent to participate. ENOS was registered (ISRCTN99414122) and approved by ethics committees/competent authorities in all participating countries. The data that support the findings of this study are available from the corresponding author on reasonable request.

### Hemodynamic Measurements

Peripheral BP and HR were measured using a validated automated monitor (Omron 705CP^27^) at the following timepoints: 3 measures at baseline (prerandomisation, day 0) and 2 on-treatment measures 1-hour postapplication of GTN patch (or at an equivalent time in the control group) on days 1 to 7. Values for minimum, mean, and maximum of the following hemodynamic derivatives were calculated:


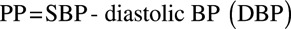



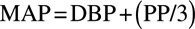



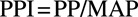



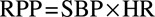


Variation in measured and derived hemodynamic parameters was assessed as

SD and

coefficient of variation=(100×SD/mean).

We chose SD as the main measure of variability because of its common use, simplicity, and relevance to clinical practice.

The recording and interpretation of peripheral BP and HR can be spurious in the setting of atrial fibrillation (AF). Therefore, in addition to analyzing the whole population, sensitivity analyses were performed excluding participants with AF.

The association between baseline hemodynamics and outcome was assessed by analyzing each baseline measure as a continuous variable. To assess the effect of GTN on between-visit BP variability, SD and coefficient of variation over days 1 to 7 were calculated for each of SBP, DBP, and MAP. In addition to analyzing these variables continuously, they were also analysed as equal quintiles with the lowest quintile as the reference group. Correlations between mean BP and BP variability over days 1 to 7 were calculated using Spearman correlation coefficient.

### Clinical Outcomes

The primary outcome of functional outcome at day 90 was measured using the 7-level mRS scale, where 0=independent and 6=dead. Secondary outcomes at day 90 included cognition: modified telephone interview for cognition scale; telephone mini-mental state examination; and verbal fluency. Patients who had died by day 90 were assigned the worst score for these outcomes. Day 90 outcomes were assessed by trained investigators, masked to treatment allocation, via telephone at national coordinating centers.

### Statistical Analysis

Data were analyzed by intention-to-treat in line with the ENOS trial statistical analysis plan^26^ and statistical analyses adopted in the primary publication.^15^ Data are number (%), median (interquartile range), or mean (SD). Baseline characteristics between groups were assessed using χ^2^ for categorical variables and 1-way ANOVA for continuous variables.

Comparisons between hemodynamics and outcome overall and between treatment groups were assessed using ANCOVA, binary logistic regression, ordinal logistic regression, or multiple linear regression. Statistical models were adjusted for prognostic baseline covariates: age, sex, baseline mRS, history of previous stroke, history of diabetes mellitus, final diagnosis, prior nitrate use, total anterior circulation syndrome, baseline Scandinavian Stroke Scale, thrombolysis, feeding status, time to randomization, and baseline SBP. Analyses involving the whole population were also adjusted for treatment allocation. The resultant odds ratio or mean difference and associated 95% CI are given, with significance set at *P*≤0.05. Odds ratios were calculated for change in hemodynamic variables of 1, 10, or 100 units as appropriate for each variable in continuous analyses. Analyses were performed using SPSS version 22 (Chicago, IL).

## Results

The ENOS trial enrolled 4011 patients with acute stroke, with mean age 70.3 (12.2) years, male sex 2297 (57.3%), severity Scandinavian Stroke Scale 33.7 (13.1), and time from onset to randomization 26 (21) hours. Those randomized to continue or stop their antihypertensives were balanced between GTN versus no GTN groups.^15^ Baseline hemodynamics did not differ between GTN and no GTN groups (Table I in the online-only Data Supplement), with overall mean BP 167.3/89.5 mm Hg and HR 77.5 bpm; 762 (19.0%) of participants had AF.

### Baseline Hemodynamics and Functional Outcome

Higher baseline values of DBP, MAP, HR, and RPP were associated with unfavorable shifts in mRS at day 90 in adjusted analyses (Table 1). However, in a sensitivity analysis excluding participants with AF, only increasing HR, RPP, and their variability were associated with unfavorable shifts in functional outcome.

**Table 1.**
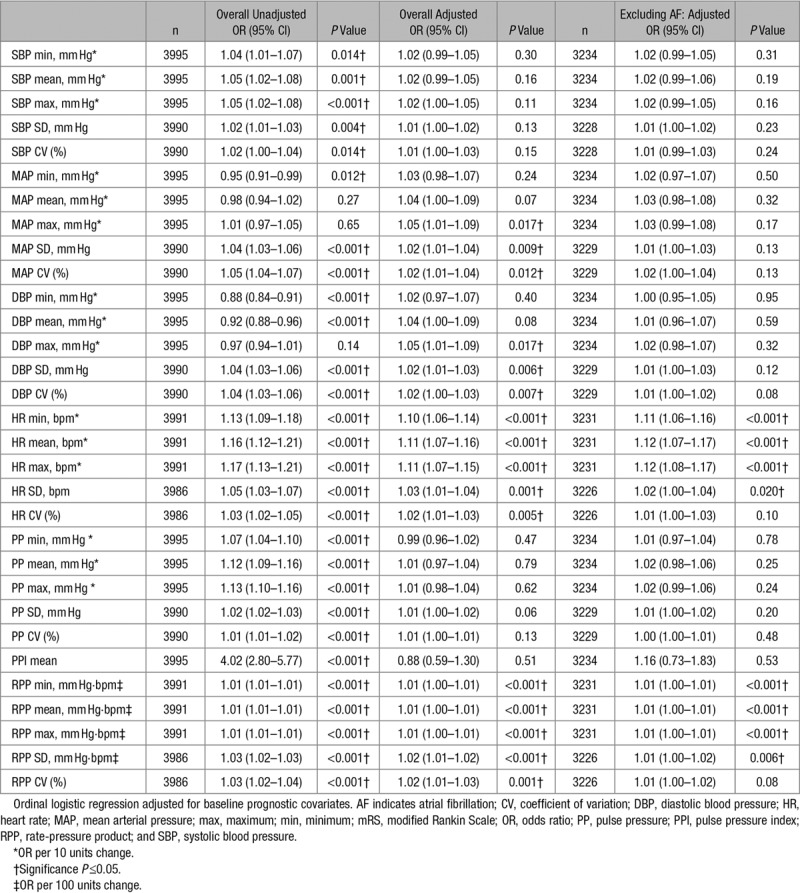
Baseline Hemodynamics Versus mRS at Day 90, Overall and Excluding Those With AF

### Between-Visit BP Variability Over Days 1 to 7 and Outcome

**Figure 1.**
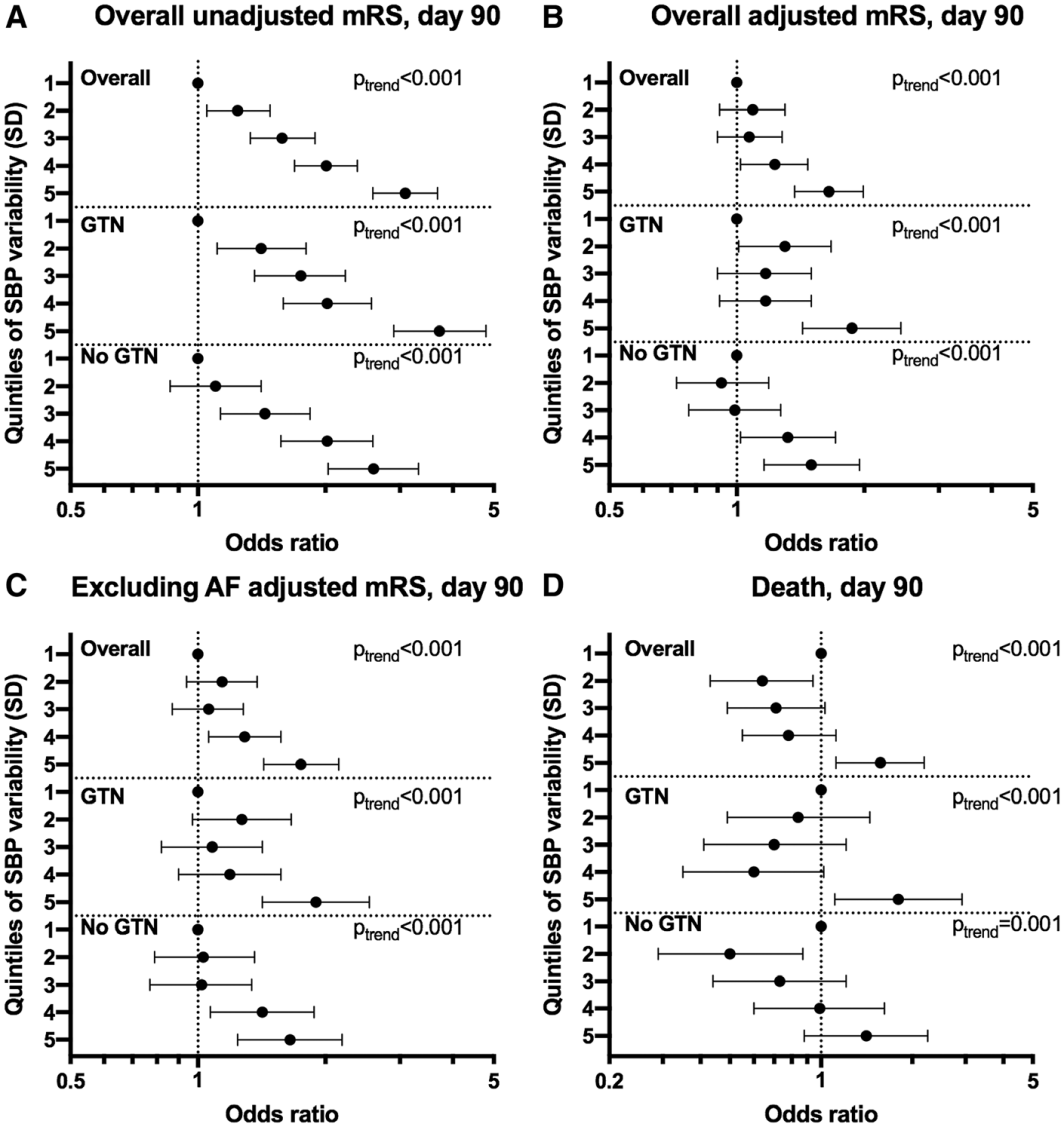
Effect of systolic blood pressure (SBP) variability over days 1 to 7 on modified Rankin Scale (mRS) and death at day 90. Quintiles of SBP variability over days 1 to 7 (reference=first quintile) vs (**A**) mRS at day 90 overall unadjusted (n=3978), (**B**) overall adjusted (n=3978), (**C**) excluding atrial fibrillation (AF) participants adjusted (n=3221), (**D**) death at day 90 overall (n=3982). Ordinal or binary logistic regression. Data are odds ratio with 95% CI. GTN indicates glyceryl trinitrate.

**Figure 2.**
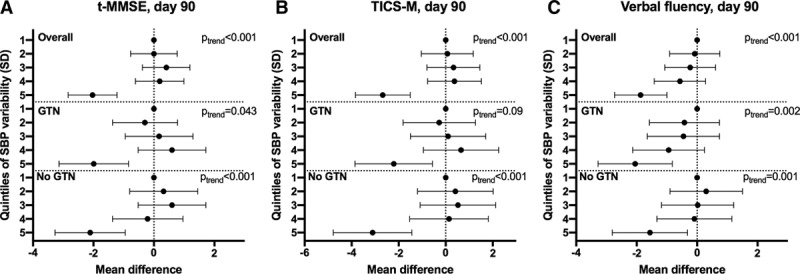
Effect of systolic blood pressure (SBP) variability over days 1 to 7 on cognition at day 90. Quintiles of SBP variability over days 1 to 7 (reference=first quintile) vs (**A**) telephone mini-mental state examination (t-MMSE; n=2019), (**B**) modified telephone interview for cognition scale (TICS-M; n=2001), (**C**) verbal fluency (n=2352). Multiple linear regression with adjustment for baseline prognostic covariates. Data are mean difference with 95% CI. GTN indicates glyceryl trinitrate.

The highest quintile of between-visit variability of SBP (SD) over days 1 to 7 was associated with an unfavourable shift in mRS (odds ratio, 1.65; 95% CI, 1.37–1.99; *P*<0.001; Figure 1) and increased risk of death at day 90 (odds ratio 1.57; 95% CI, 1.12–2.19; *P*=0.009). These associations were maintained when participants with AF were excluded and when analyzed as a continuous variable. Similarly, the highest quintile was also associated with worse cognitive scores at day 90 compared with the lowest quintile: telephone mini-mental state examination mean difference, −2.03; 95% CI, −2.84 to −1.22; *P*<0.001; modified telephone interview for cognition scale mean difference, −2.68; 95% CI −3.84 to −1.51; *P*<0.001; verbal fluency mean difference, −1.87; 95% CI, −2.73 to −1.00; *P*<0.001 (Figure 2). These associations were maintained after excluding participants with AF. Furthermore, analogous associations with these outcomes were seen across all measures of between-visit BP variability for SBP (Figure I in the online-only Data Supplement), DBP (Figures II and III in the online-only Data Supplement), and MAP (Figures IV and V in the online-only Data Supplement).

Mean SBP over days 1 to 7 was weakly correlated with measures of between-visit SBP variability (Spearman correlation coefficient: SD, 0.137; coefficient of variation, −0.155). Similar weak correlations between mean DBP and MAP and their corresponding measures of variability were seen. In addition, there was no correlation between within-individual BP trend over the treatment period and mRS at day 90, highlighting that the associations between variability and outcome seen were not mediated by trend in mean BP over time.

### Hemodynamic Effects of GTN

Table 2 shows the effect of GTN versus no GTN on hemodynamic parameters at day 1 that is, on treatment (n=3851, 96%). Overall, GTN lowered mean BP by 7/3.6 mm Hg, MAP by 4.7 mm Hg, and PP by 3.4 mm Hg. GTN increased HR by 1.4 bpm but reduced RPP (the product of SBP and HR). GTN had no effect on within-visit variability of any hemodynamic variable at day 1. The hemodynamic effects of GTN were maintained in those without AF (Table II in the online-only Data Supplement). GTN lowered mean BP by 6.8/3.4 mm Hg in ischemic stroke patients and by 7.5/4.9 mm Hg in those with ICH at day 1 and reduced within-visit variability of SBP (SD) at day 1 by 1.3 mm Hg in those with ICH, but not in ischemic stroke.

**Table 2.**
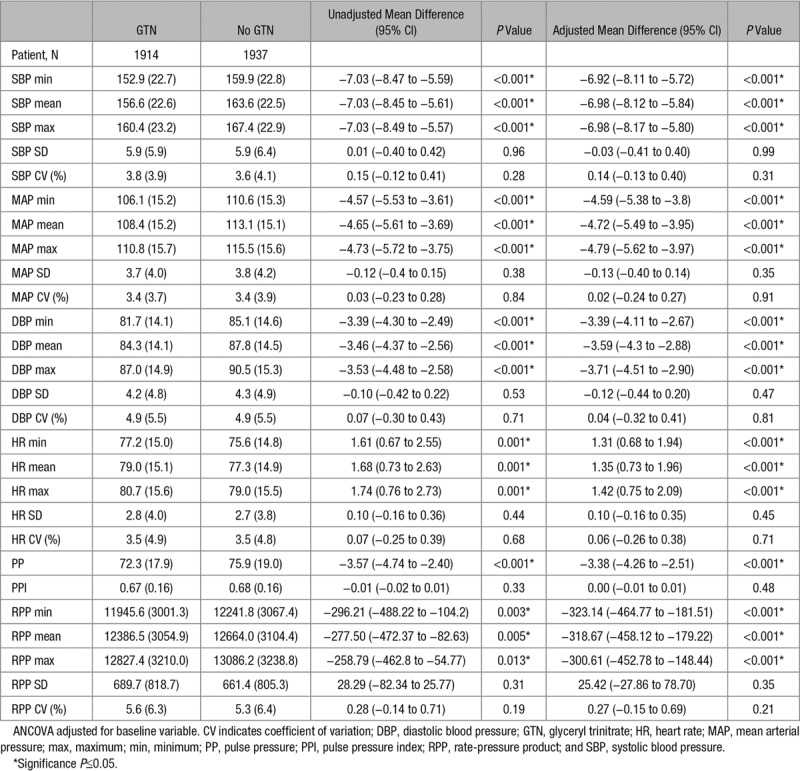
Effect of GTN Versus No GTN on Hemodynamic Variables at Day 1

Over 7 days of treatment, GTN lowered mean BP by 2.9/2.1 mm Hg, MAP by 2.3 mm Hg, PP by 0.8 mm Hg, and increased HR by 1 bpm on average (Table III in the online-only Data Supplement). These results are dampened in comparison to the day 1 data (Table 2), highlighting tachyphylaxis seen with GTN over time. GTN lowered between-visit variability of SBP (SD) over days 1 to 7 by 0.4 mm Hg overall and by 1.1 mm Hg in those with ICH. In contrast, GTN did not change SBP coefficient of variation or between-visit variability of DBP. These effects were maintained in those without AF (Table IV in the online-only Data Supplement).

## Discussion

In this prespecified secondary analysis of ENOS trial data, baseline hemodynamic parameters were associated with worse functional outcome at day 90. Increased between-visit BP variability over days 1 to 7 was associated with worse functional and cognitive outcomes and increased odds of death at day 90. GTN lowered BP and RPP despite increasing HR at day 1 and reduced between-visit variability of SBP over days 1 to 7.

Several secondary analyses of large trials have demonstrated associations between both high and low BP and poor clinical outcomes after acute ischemic stroke.^2,8,28^ Although we found no association between baseline SBP and functional outcome, increased maximum DBP and MAP were both independently associated with an unfavorable shift in mRS at day 90. Although 1 cohort found that baseline MAP—and not DBP—was associated with death or dependency at 90 days,^8^ others have reported no effect of DBP on early outcomes at day 10.^9^

All derived measures of higher baseline HR and RPP were associated with an unfavorable shift in mRS at day 90, both overall and in those without AF. High baseline HR is associated with increased death, heart failure and dependency at 90 days in both acute ischemic and hemorrhagic stroke.^6,29,30^ High baseline HR is a surrogate for clinical frailty and comorbidity burden^31^ but may also represent underlying dehydration, anemia and stroke severity, which are all independently associated with poor prognosis after stroke.^32,33^ Although there are fewer data pertaining to RPP in acute stroke, increased baseline RPP has been associated with death or dependency at day 90.^8^ In addition to confirming this finding, we demonstrated that increased within-visit variability of RPP at baseline was associated with an unfavorable shift in mRS, a novel finding in acute stroke.

Increased between-visit variability of SBP over days 1 to 7 was associated with an unfavorable shift in mRS, worse cognitive scores, and increased odds of death at day 90 independent of trend in mean BP over time. Therefore, fluctuations in BP in the days after stroke may have a greater influence on 90-day outcome than absolute mean BP; in line with a recently reported analysis in ICH patients.^34^ We add to the growing body of evidence that increased between-visit BP variability (SBP, DBP, and MAP) is associated with poor clinical outcome after acute stroke.^7,10^ Furthermore, we report novel associations with increased between-visit BP variability and worse cognitive scores at 90 days across 3 cognitive domains.

Using data from one of the largest BP-lowering trials in acute stroke, we have confirmed transdermal GTN’s aforementioned effects on hemodynamics.^21^ The significant reduction in RPP at day 1, implies that GTN’s ability to increase HR is negated by its BP-lowering effect. GTN significantly lowered between-visit variability of SBP over days 1 to 7, mirroring a similar finding in a pooled analysis of 4 GTN pilot studies.^35^ Whether this modest reduction is sufficient to impact upon clinical outcome requires further testing.

The timing of hemodynamic measurements in relation to stroke onset is important. The maintenance of cerebral blood flow through autoregulation is impaired in acute stroke with cerebral perfusion pressure becoming dependent on systemic BP.^36^ Potentially viable brain tissue may, therefore, be sensitive to greater variability in BP with peaks increasing the risk of hemorrhagic transformation in ischemic stroke or hematoma expansion in ICH, and cerebral edema in both stroke types, whereas troughs cause further ischemic injury.^5,10^ If so, then medications that lower BP variability may be best assessed as early as possible after stroke onset before these effects manifest. Although this potential time-dependent mechanism is speculative, it may be 1 way in which GTN may exert its apparent beneficial impact on clinical outcomes.^35^ Furthermore, GTN reduces arterial stiffness,^35^ which is independently associated with hemorrhagic transformation^37^ and poor collateral status^38^ in acute ischemic stroke. The efficacy of transdermal GTN given within 4 hours of onset is currently being assessed in the large RIGHT-2 (Rapid Intervention With Glyceryl Trinitrate in Hypertensive Stroke Trial 2).^39^

The strengths of the present study include: its large sample size using data from one of the largest BP trials in acute stroke; the use of prespecified analyses and ordinal logistic regression to increase power compared with binary analysis of mRS; and generalisability with analyses including the vast majority of recruited participants from a variety of countries, stroke services, and patient populations.

This study has several limitations. First, some comparisons were observational, and the results should be interpreted with caution because of the inherent risk of confounding. Despite adjusting for baseline prognostic factors, we cannot exclude reverse causality, for example, if patients with larger, more severe strokes also had increased BP variability. Second, the inclusion criterion of SBP 140 to 220 mm Hg systematically excluded patients and thus reduces generalisability, although this is likely to have attenuated rather than enhanced the strength of reported associations. Third, although a validated automated monitor was used for measurements, beat-to-beat data were not available, limiting the ability to detect within-visit variability. Fourth, a minority of patients had AF which can reduce the accuracy of hemodynamic measurements; however, sensitivity analyses excluding patients with AF were performed to account for this. Fifth, cognitive outcome data were only available for around 50% of participants, and their analyses may have lacked power, weakening the observed associations but given the severity of the stroke population recruited this is inevitable. Lastly, participants were recruited a median of 26 hours after stroke onset: longer than previous studies that have detected associations between hemodynamic measures and functional outcome.^10^

Smooth lowering of elevated BP with avoidance of peaks and troughs over the first days after stroke should be considered by clinicians in both acute ischemic and hemorrhagic stroke. Whether smooth and sustained BP control is beneficial has yet to be tested directly in randomized controlled trials. If BP variability is a modifiable target in acute stroke, then agents that lower it, including GTN, may be of benefit.

## Acknowledgments

We thank the participants, investigators, and research staff involved in the ENOS trial (Efficacy of Nitric Oxide in Stroke). J.P. Appleton wrote the first draft and performed the analyses. P.M. Bath conceived the study, amended the article, and is the project guarantor. All authors commented on and approved the article.

## Sources of Funding

ENOS (Efficacy of Nitric Oxide in Stroke) was funded by the British United Provident Association UK Foundation and Medical Research Council (G0501797). J.P. Appleton is funded by National Institute for Health Research TARDIS (10/104/24) trial (Triple Antiplatelets for Reducing Dependency after Ischaemic Stroke) and British Heart Foundation RIGHT-2 (Rapid Intervention With Glyceryl Trinitrate in Hypertensive Stroke Trial 2; CS/14/4/30972).

## Disclosures

P.M. Bath is Stroke Association Professor of Stroke Medicine and Chief Investigator of the ENOS trial (Efficacy of Nitric Oxide in Stroke) and RIGHT-2 (Rapid Intervention With Glyceryl Trinitrate in Hypertensive Stroke Trial 2). P.M. Bath and P.M. Rothwell are National Institute for Health Research Senior Investigators. The other authors report no conflicts

## Supplementary Material
