# Supplementary material for: Effect of Glyceryl Trinitrate on Hemodynamics in Acute Stroke: Data From the ENOS Trial
Source: Stroke. 2019 Jan 10;50(2):405–12. doi: 10.1161/STROKEAHA.118.023190 (PMC6358219; doi:10.1161/STROKEAHA.118.023190)
Supplement: Supplementary file 1 [file str-50-405-s001.pdf]

SUPPLEMENTARY MATERIAL

Effect of glyceryl trinitrate on haemodynamics in acute stroke: data from the Efficacy of Nitric Oxide in Stroke trial

1. Efficacy of Nitric Oxide in Stroke (ENOS) trial steering and international advisory committees 2
2. Supplementary tables 3
3. Supplementary figures 10

1. **Efficacy of Nitric Oxide in Stroke (ENOS) trial steering and international advisory committees**

Trial Steering Committee:
D Thomas (Independent Chair to 2006, UK), G Venables (Independent Chair from 2006, UK), P Amarenco (Independent Physician, France), K Muir (Independent Physician, UK), P M W Bath (Chief Investigator, UK), N Sprigg (Deputy Chief Investigator, UK), E Berge (Norway), K R Lees (UK), S Pocock (Statistician from 2003, UK), A Shone (Sponsor's Representative, UK), A Skene (Statistician to 2003, UK), J M Wardlaw (Neuroradiologist, UK), D Whynes (Health economist, UK).

International Advisory Committee:
P M W Bath (Chair, UK), E Berge (Norway), M Beridze (Georgia), C Bladin (Australia), V Caso (Italy), C Chen/H M Chang (Singapore), H Christensen (Denmark), R Collins (Eire), A Czlonkowska (Poland), E Díez-Tejedor (Spain), A El Etribi (Egypt), A R Ghani (Malaysia), J Gommans (New Zealand), A C Laska (Sweden), K R Lees (UK), J Navarro (Philippines), G Ntaios (Greece), S Ozturk (Turkey), S Phillips (Canada), K Prasad (India), H A de Silva (Sri Lanka), S Szatmari (Romania), L Wong (Hong Kong), Y-J Wang (China).

2. Supplementary tables

Supplementary Table I: Baseline haemodynamics GTN vs. no GTN

	Overall	GTN	No GTN
Patients (N)	4011	2000	2011
SBP min (mmHg)	160.3 (19.4)	160.4 (19.3)	160.3 (19.5)
SBP mean (mmHg)	167.3 (19.0)	167.4 (18.8)	167.1 (19.2)
SBP max (mmHg)	174.2 (20.0)	174.3 (19.7)	174.0 (20.3)
SBP SD (mmHg)	7.3 (5.6)	7.3 (5.7)	7.3 (5.4)
SBP CoV (%)	4.4 (3.5)	4.4 (3.6)	4.4 (3.3)
MAP min (mmHg)	111.1 (13.1)	111.3 (13.1)	111.0 (13.2)
MAP mean (mmHg)	115.4 (13.1)	115.6 (12.9)	115.2 (13.2)
MAP max (mmHg)	119.9 (14.0)	120.0 (13.7)	119.8 (14.3)
MAP SD (mmHg)	4.6 (3.7)	4.6 (3.8)	4.6 (3.7)
MAP CoV (%)	4.0 (3.2)	4.0 (3.3)	4.0 (3.2)
DBP min (mmHg)	84.7 (13.1)	84.8 (13.1)	84.6 (13.2)
DBP mean (mmHg)	89.5 (13.1)	89.7 (13.0)	89.4 (13.3)
DBP max (mmHg)	94.6 (14.6)	94.8 (14.3)	94.4 (14.8)
DBP SD (mmHg)	5.3 (4.5)	5.3 (4.6)	5.2 (4.5)
DBP CoV (%)	5.9 (5.0)	5.9 (5.1)	5.8 (5.0)
HR min (bpm)	74.5 (14.5)	74.7 (14.6)	74.3 (14.4)
HR mean (bpm)	77.5 (14.7)	77.7 (14.8)	77.3 (14.6)
HR max (bpm)	80.6 (15.7)	80.8 (15.8)	80.4 (15.7)
HR SD (bpm)	3.3 (3.7)	3.3 (3.7)	3.2 (3.6)
HR CoV (%)	4.2 (4.5)	4.2 (4.6)	4.2 (4.4)
PP (mmHg)	77.7 (16.9)	77.7 (16.9)	77.8 (17.0)
PPI	0.68 (0.15)	0.68 (0.15)	0.68 (0.15)
RPP min (mmHg.bpm)	12163.1 (2787.6)	12183.5 (2758.1)	12142.9 (2817.1)
RPP mean (mmHg.bpm)	12968.1 (2884.8)	12998.6 (2859.2)	12937.8 (2910.5)
RPP max (mmHg.bpm)	13782.8 (3153.7)	13817.0 (3123.4)	13748.7 (3183.8)
RPP SD (mmHg.bpm)	853.9 (767.1)	861.4 (778.2)	846.5 (756.1)
RPP CoV (%)	6.6 (5.5)	6.6 (5.6)	6.5 (5.4)

Data are number (%) or mean (standard deviation [SD]). BP: blood pressure; CoV: coefficient of variation; DBP: diastolic BP; HR: heart rate; MAP: mean arterial pressure; PP: pulse pressure; PPI: pulse pressure index; RPP: rate pressure product; SBP: systolic BP.

Supplementary Table II: Effect of GTN vs. no GTN on haemodynamic variables at day 1 excluding those with AF

	GTN	No GTN	Unadjusted Mean Difference (95% CI)	p	Adjusted Mean Difference (95% CI)	p
Patients (N)	1534	1582				
SBP min	153.1 (22.9)	160.4 (23.2)	-7.34 (-8.96, -5.72)	**<0.001**	-7.10 (-8.43, -5.77)	**<0.001**
SBP mean	156.7 (22.9)	164.1 (22.9)	-7.37 (-8.97, -5.76)	**<0.001**	-7.14 (-8.42, -5.86)	**<0.001**
SBP max	160.3 (23.4)	167.7 (23.4)	-7.39 (-9.03, -5.75)	**<0.001**	-7.14 (-8.47, -5.80)	**<0.001**
SBP SD	5.6 (5.6)	5.7 (6.3)	-0.10 (-0.54, 0.34)	0.66	-0.11 (-0.55, 0.33)	0.62
SBP CV (%)	3.6 (3.6)	3.5 (4.1)	0.07 (-0.21, 0.36)	0.61	0.07 (-0.22, 0.35)	0.65
MAP min	105.9 (15.5)	110.7 (15.4)	-4.82 (-5.91, -3.74)	**<0.001**	-4.77 (-5.65, -3.89)	**<0.001**
MAP mean	108.1 (15.4)	113.0 (15.4)	-4.88 (-5.96, -3.80)	**<0.001**	-4.84 (-5.71, -3.99)	**<0.001**
MAP max	110.4 (15.9)	115.3 (15.8)	-4.93 (-6.05, -3.82)	**<0.001**	-4.85 (-5.77, -3.92)	**<0.001**
MAP SD	3.5 (3.9)	3.6 (4.2)	-0.12 (-0.42, 0.18)	0.42	-0.13 (-0.42, 0.17)	0.41
MAP CV (%)	3.3 (3.6)	3.2 (3.7)	0.04 (-0.23, 0.31)	0.77	0.03 (-0.24, 0.30)	0.81
DBP min	81.3 (14.2)	85.0 (14.6)	-3.64 (-4.66, -2.63)	**<0.001**	-3.64 (-4.44, -2.84)	**<0.001**
DBP mean	83.9 (14.2)	87.5 (14.6)	-3.64 (-4.65, -2.62)	**<0.001**	-3.69 (-4.48, -2.90)	**<0.001**
DBP max	86.4 (14.9)	90.0 (15.3)	-3.63 (-4.69, -2.56)	**<0.001**	-3.70 (-4.59, -2.81)	**<0.001**
DBP SD	3.9 (4.8)	4.0 (4.6)	-0.03 (-0.37, 0.32)	0.88	-0.12 (-0.44, 0.20)	0.47
DBP CV (%)	4.7 (5.5)	4.5 (5.2)	0.18 (-0.21, 0.57)	0.37	0.16 (-0.23, 0.55)	0.41
HR min	76.2 (14.6)	74.8 (14.5)	1.44 (0.41, 2.46)	**0.006**	1.35 (0.68, 2.02)	**<0.001**
HR mean	77.8 (14.6)	76.3 (14.6)	1.48 (0.45, 2.51)	**0.005**	1.38 (0.73, 2.04)	**<0.001**
HR max	79.3 (14.9)	77.8 (15.1)	1.53 (0.47, 2.58)	**0.005**	1.40 (0.69, 2.11)	**<0.001**
HR SD	2.4 (3.2)	2.4 (3.6)	0.04 (-0.21, 0.29)	0.76	0.04 (-0.21, 0.29)	0.76
HR CV (%)	3.1 (4.2)	3.1 (4.5)	0.03 (-0.29, 0.36)	0.85	0.02 (-0.30, 0.34)	0.90
PP	72.8 (17.7)	76.6 (19.1)	-3.73 (-5.02, -2.44)	**<0.001**	-3.44 (-4.42, -2.47)	**<0.001**
PPI	0.68 (0.15)	0.68 (0.16)	-0.01 (-0.02, 0.01)	0.41	0.00 (-0.01, 0.01)	0.61
RPP min	11811.1 (2990.1)	12143.8 (3057.3)	-232.57 (-545.37, -119.77)	**0.002**	-314.19 (-467.97, -160.41)	**<0.001**
RPP mean	12219.5 (3031.9)	12536.7 (3097.8)	-317.26 (-532.96, -101.56)	**0.004**	-305.64 (-456.26, -155.01)	**<0.001**
RPP max	12627.7 (3150.6)	12929.7 (3232.2)	-301.95 (-526.57, -77.34)	**0.008**	-290.21 (-453.97, -126.46)	**0.001**
RPP SD	632.4 (701.9)	617.1 (793.4)	15.34 (-40.04, 70.72)	0.59	13.43 (-41.48, 68.33)	0.63

RPP CV (%)	5.2 (5.8)	5.0 (6.3)	0.24 (-0.21, 0.69)	0.30	0.22 (-0.23, 0.66)	0.33

Analysis of covariance (ANCOVA) with adjustment for baseline variable. AF: atrial fibrillation; BP: blood pressure; CoV: coefficient of variation; DBP: diastolic BP; HR: heart rate; MAP: mean arterial pressure; PP: pulse pressure; PPI: pulse pressure index; RPP: rate pressure product; SBP: systolic BP; SD: standard deviation.

Supplementary Table III: Effect of GTN vs. no GTN on haemodynamic variables over days 1 to 7 overall

	GTN	No GTN	Unadjusted Mean Difference (95% CI)	p	Adjusted Mean Difference (95% CI)	p
Patients (N)	1996	2001				
SBP min	129.2 (20.1)	131.3 (20.2)	-2.14 (-3.39, -0.89)	**0.001**	-2.25 (-3.38, -1.11)	**<0.001**
SBP mean	153.3 (19.0)	156.0 (18.8)	-2.71 (-3.88, -1.54)	**<0.001**	-2.87 (-3.81, -1.91)	**<0.001**
SBP max	178.3 (22.9)	181.6 (22.5)	-3.31 (-4.71, -1.90)	**<0.001**	-3.48 (-4.64, -2.32)	**<0.001**
SBP SD	15.5 (6.2)	15.9 (6.4)	-0.38 (-0.77, 0.01)	0.056	-0.40 (-0.78, -0.02)	**0.041**
SBP CV (%)	10.2 (4.1)	10.3 (4.2)	-0.08 (-0.34, 0.18)	0.53	-0.08 (-0.34, 0.17)	0.52
MAP min	91.0 (13.6)	93.1 (13.7)	-2.05 (-2.90, -1.20)	**<0.001**	-2.21 (-2.95, -1.46)	**<0.001**
MAP mean	106.9 (12.6)	109.1 (12.7)	-2.15 (-2.93, -1.37)	**<0.001**	-2.33 (-2.96, -1.71)	**<0.001**
MAP max	123.8 (15.0)	126.0 (15.2)	-2.28 (-3.22, -1.35)	**<0.001**	-2.48 (-3.27, -1.70)	**<0.001**
MAP SD	10.4 (4.1)	10.4 (4.2)	-0.02 (-0.28, 0.24)	0.89	-0.03 (-0.29, 0.22)	0.81
MAP CV (%)	9.8 (3.9)	9.6 (3.9)	0.17 (-0.07, 0.42)	0.17	0.17 (-0.07, 0.42)	0.16
DBP min	69.0 (11.9)	70.9 (12.3)	-1.95 (-2.70, -1.20)	**<0.001**	-2.12 (-2.75, -1.48)	**<0.001**
DBP mean	83.8 (11.2)	85.6 (11.8)	-1.87 (-2.59, -1.16)	**<0.001**	-2.07 (-2.61, -1.52)	**<0.001**
DBP max	100.3 (14.5)	102.2 (14.6)	-1.97 (-2.87, -1.07)	**<0.001**	-2.16 (-2.94, -1.39)	**<0.001**
DBP SD	9.7 (3.9)	9.7 (3.9)	0.01 (-0.24, 0.25)	0.97	0.00 (-0.25, 0.24)	0.97
DBP CV (%)	11.7 (4.7)	11.5 (4.6)	0.24 (-0.05, 0.53)	0.10	0.26 (-0.03, 0.54)	0.08
HR min	66.1 (12.0)	65.2 (11.4)	0.97 (0.24, 1.69)	**0.009**	0.82 (0.23, 1.41)	**0.006**
HR mean	78.2 (12.4)	77.1 (12.0)	1.12 (0.36, 1.88)	**0.004**	0.93 (0.38, 1.49)	**0.001**
HR max	92.2 (16.7)	90.9 (16.9)	1.22 (0.18, 2.26)	**0.020**	1.01 (0.15, 1.87)	**0.020**
HR SD	8.4 (4.7)	8.3 (4.6)	0.07 (-0.22, 0.36)	0.63	0.06 (-0.23, 0.34)	0.70
HR CV (%)	10.7 (5.6)	10.7 (5.5)	-0.01 (-0.36, 0.33)	0.95	-0.01 (-0.36, 0.33)	0.94
PP	69.5 (14.6)	70.4 (14.8)	-0.84 (-1.75, 0.08)	0.07	-0.79 (-1.48, -0.10)	**0.025**
PPI	0.65 (0.12)	0.65 (0.13)	0.00 (0.00, 0.01)	0.28	0.01 (0.00, 0.01)	**0.034**
RPP min	9361.5 (2225.5)	9368.4 (2164.7)	-7.00 (-143.16, 129.19)	0.92	-29.78 (-145.93, 86.37)	0.62
RPP mean	11984.6 (2417.4)	12028.3 (2411.9)	-43.73 (-193.5, 106.05)	0.57	-78.39 (-193.08, 36.30)	0.18
RPP max	15090.2 (3398.3)	15195.5 (3502.7)	-105.31 (-319.37, 108.75)	0.34	-152.13 (-325.96, 21.71)	0.09
RPP SD	1805.8 (874.1)	1841.8 (908.1)	-36.01 (-91.33, 19.30)	0.20	-42.45 (-94.99, 10.10)	0.11

RPP CV (%)	15.1 (6.7)	15.3 (6.7)	-0.16 (-0.58, 0.25)	0.44	-0.18 (-0.59, 0.23)	0.39

Data mean (SD) or mean difference (95% confidence intervals). Analysis of covariance (ANCOVA) with adjustment for baseline variable. BP: blood pressure; CoV: coefficient of variation; DBP: diastolic BP; HR: heart rate; MAP: mean arterial pressure; PP: pulse pressure; PPI: pulse pressure index; RPP: rate pressure product; SBP: systolic BP; SD: standard deviation.

Supplementary Table IV: Effect of GTN vs. no GTN on haemodynamic variables over days 1 to 7 excluding those with AF

	GTN	No GTN	Unadjusted Mean Difference (95% CI)	p	Adjusted Mean Difference (95% CI)	p
Patients (N)	1602	1636				
SBP min	130.7 (20.1)	132.4 (20.3)	-1.74 (-3.13, -0.34)	**0.015**	-1.75 (-3.00, -0.50)	**0.006**
SBP mean	154.2 (19.3)	156.6 (19.3)	-2.40 (-3.73, -1.07)	**<0.001**	-2.43 (-3.50, -1.37)	**<0.001**
SBP max	178.9 (23.3)	181.8 (23.1)	-2.92 (-4.52, -1.32)	**<0.001**	-2.92 (-4.24, -1.61)	**<0.001**
SBP SD	15.2 (6.2)	15.6 (6.3)	-0.43 (-0.86, 0.05)	0.053	-0.43 (-0.85, -0.01)	**0.046**
SBP CV (%)	9.9 (4.0)	10.1 (4.0)	-0.14 (-0.41, 0.14)	0.34	-0.13 (-0.41, 0.15)	0.35
MAP min	91.8 (13.6)	93.6 (13.8)	-1.78 (-2.73, -1.84)	**<0.001**	-1.87 (-2.69, -1.04)	**<0.001**
MAP mean	107.2 (12.8)	109.1 (12.9)	-1.94 (-2.83, -1.05)	**<0.001**	-2.05 (-2.74, -1.35)	**<0.001**
MAP max	123.8 (15.3)	125.7 (15.4)	-1.96 (-3.02, -0.90)	**<0.001**	-2.01 (-2.89, -1.12)	**<0.001**
MAP SD	10.1 (4.1)	10.2 (4.1)	-0.05 (-0.33, 0.23)	0.89	-0.04 (-0.32, 0.24)	0.77
MAP CV (%)	9.5 (3.8)	9.4 (3.8)	0.11 (-0.15, 0.38)	0.41	0.11 (-0.15, 0.38)	0.40
DBP min	69.4 (12.0)	71.2 (12.4)	-1.77 (-2.61, -0.93)	**<0.001**	-1.86 (-2.56, -1.17)	**<0.001**
DBP mean	83.7 (11.4)	85.4 (11.8)	-1.71 (-2.51, -0.91)	**<0.001**	-1.85 (-2.45, -1.25)	**<0.001**
DBP max	99.8 (14.7)	101.6 (14.7)	-1.82 (-2.83, -0.81)	**<0.001**	-1.94 (-2.81, -1.06)	**<0.001**
DBP SD	9.4 (3.9)	9.4 (3.8)	-0.04 (-0.31, 0.22)	0.75	-0.04 (-0.31, 0.22)	0.76
DBP CV (%)	11.3 (4.7)	11.2 (4.6)	0.15 (-0.17, 0.47)	0.35	0.15 (-0.17, 0.46)	0.36
HR min	65.7 (11.5)	64.9 (11.1)	0.82 (0.04, 1.60)	**0.039**	0.80 (0.20, 1.41)	**0.010**
HR mean	77.0 (11.8)	76.1 (11.7)	0.97 (0.16, 1.78)	**0.019**	0.94 (0.36, 1.53)	**0.002**
HR max	90.3 (15.5)	89.3 (16.0)	0.99 (-0.10, 2.07)	0.07	0.95 (0.04, 1.85)	**0.040**
HR SD	7.9 (4.3)	7.9 (4.2)	0.03 (-0.27, 0.32)	0.86	0.03 (-0.27, 0.32)	0.85
HR CV (%)	10.3 (5.2)	10.3 (5.2)	-0.05 (-0.41, 0.31)	0.80	-0.05 (-0.41, 0.30)	0.77
PP	70.4 (14.5)	71.1 (15.0)	-0.69 (-1.71, 0.33)	0.18	-0.59 (-1.35, -0.18)	0.13
PPI	0.66 (0.12)	0.65 (0.13)	0.01 (0.00, 0.01)	0.28	0.01 (0.00, 0.01)	**0.033**
RPP min	9393.1 (2195.1)	9381.8 (2121.9)	11.31 (-137.44, 160.07)	0.88	16.95 (-107.39, 141.28)	0.79
RPP mean	11884.0 (2380.8)	11916.9 (2394.0)	-32.89 (-197.42, 131.65)	0.70	-33.27 (-155.89, 89.34)	0.60
RPP max	14854.3 (3296.5)	14954.8 (3454.4)	-100.47 (-333.22, 132.28)	0.40	-102.87 (-291.00, 85.26)	0.28

RPP SD	1717.6 (817.1)	1761.9 (855.8)	-44.29 (-101.98, 13.40)	0.13	-44.55 (-101.76, 12.65)	0.13
RPP CV (%)	14.5 (6.2)	14.7 (6.3)	-0.24 (-0.68, 0.19)	0.27	-0.25 (-0.68, 0.18)	0.26

Data mean (SD) or mean difference (95% confidence intervals). Analysis of covariance (ANCOVA) with adjustment for baseline variable. AF: atrial fibrillation; BP: blood pressure; CoV: coefficient of variation; DBP: diastolic BP; HR: heart rate; MAP: mean arterial pressure; PP: pulse pressure; PPI: pulse pressure index; RPP: rate pressure product; SBP: systolic BP; SD: standard deviation.

3. Supplementary Figures

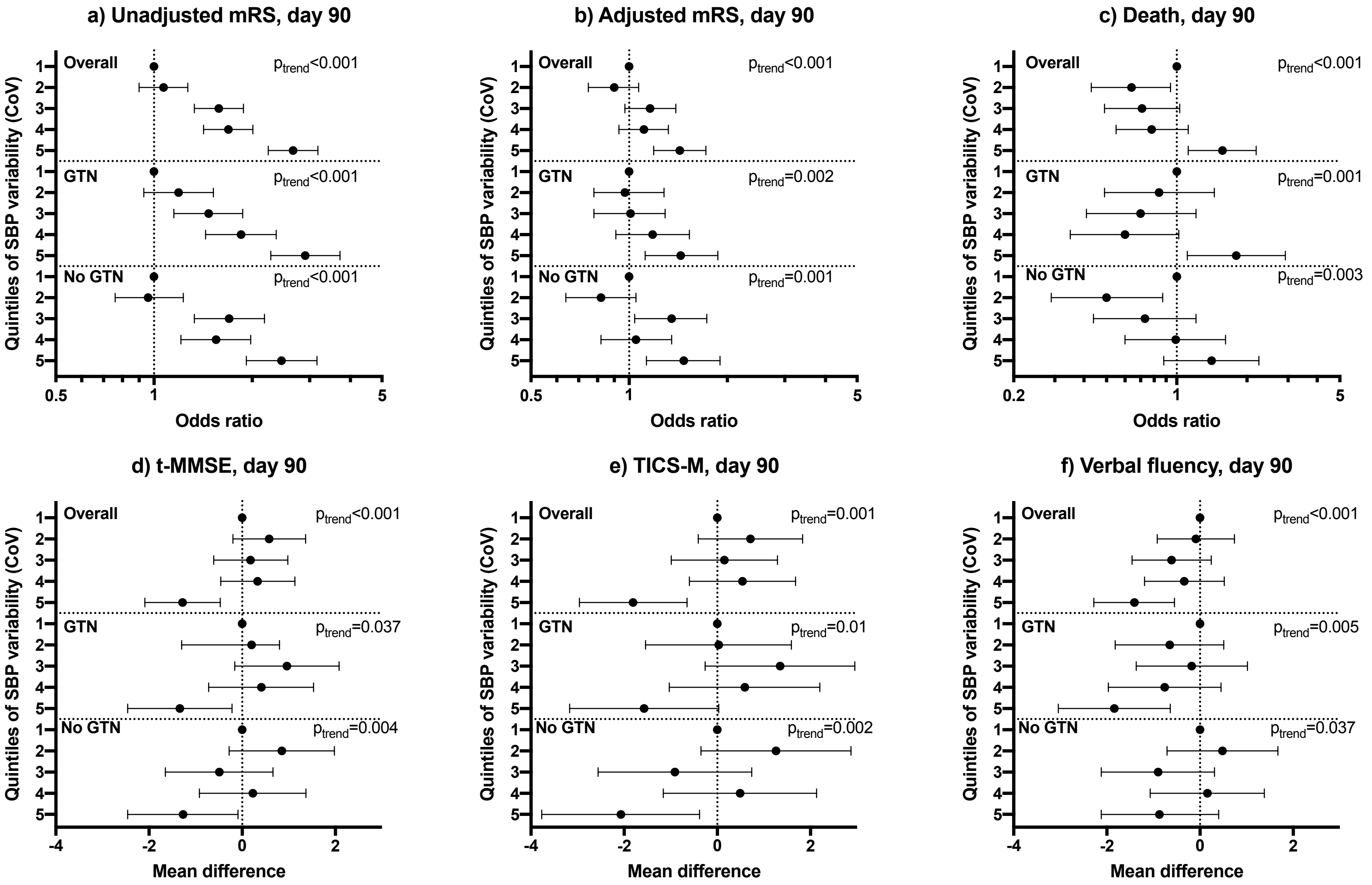

Supplementary Figure I: Effect of SBP variability over days 1 to 7 (CoV) on day 90 outcomes
a-b: Quintiles of SBP variability over days 1 to 7 (CoV) vs. mRS at day 90 overall and in GTN and no GTN groups, with the first quintile as reference, (a) overall unadjusted (n=3978), (b) overall adjusted (n=3978).
c: Quintiles of SBP variability over days 1 to 7 (CoV) vs. death at day 90 overall (n=3982) and in GTN and no GTN groups, with the first quintile as reference.
d-f: Quintiles of SBP variability over days 1 to 7 (CoV) vs. cognitive outcomes at day 90 overall and in GTN and no GTN groups, with the first quintile as reference, (d) t-MMSE (n=2019), (e) TICS-M (n=2001), (f) verbal fluency (n=2352).
Ordinal (a-b) or binary (c) logistic or multiple linear (d-f) regression with adjustment for age, sex, baseline mRS, history of previous stroke, history of diabetes, final diagnosis, prior nitrate use, baseline SSS, total anterior circulation stroke, thrombolysis, feeding status, time to randomisation, baseline SBP, GTN vs no GTN, continue/stop. Data are odds ratios or mean difference with 95% confidence intervals. AF: atrial fibrillation; CoV: coefficient of variation; GTN: glyceryl trinitrate; mRS: modified Rankin Scale; SBP: systolic blood pressure; SSS: Scandinavian Stroke Scale; t-MMSE: telephone mini-mental state examination; TICS-M: modified telephone interview for cognition scale.

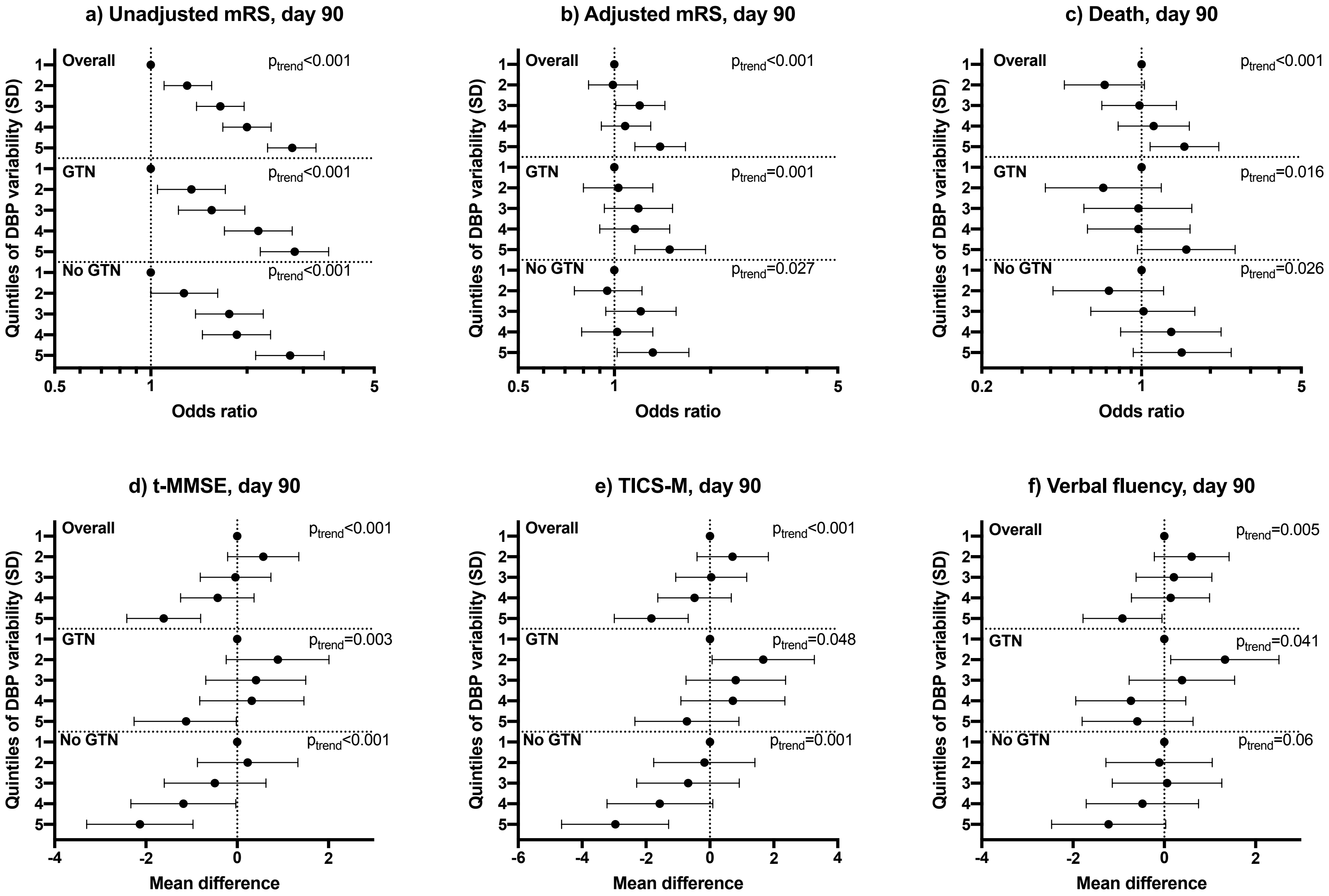

a) Unadjusted mRS, day 90
b) Adjusted mRS, day 90
c) Death, day 90
d) t-MMSE, day 90
e) TICS-M, day 90
f) Verbal fluency, day 90
Quintiles of DBP variability (SD)
Overall
GTN
No GTN
Odds ratio
Mean difference

Supplementary Figure II: Effect of DBP variability over days 1 to 7 (SD) on day 90 outcomes
a-b: Quintiles of DBP variability over days 1 to 7 (SD) vs. mRS at day 90 overall and in GTN and no GTN groups, with the first quintile as reference, (a) overall unadjusted (n=3978), (b) overall adjusted (n=3978).
c: Quintiles of DBP variability over days 1 to 7 (SD) vs. death at day 90 overall (n=3982) and in GTN and no GTN groups, with the first quintile as reference.
d-f: Quintiles of DBP variability over days 1 to 7 (SD) vs. cognitive outcomes at day 90 overall and in GTN and no GTN groups, with the first quintile as reference, (d) t-MMSE (n=2019), (e) TICS-M (n=2001), (f) verbal fluency (n=2352).
Ordinal (a-b) or binary (c) logistic or multiple linear (d-f) regression with adjustment for age, sex, baseline mRS, history of previous stroke, history of diabetes, final diagnosis, prior nitrate use, baseline SSS, total anterior circulation stroke, thrombolysis, feeding status, time to randomisation, baseline SBP, GTN vs no GTN, continue/stop. Data are odds ratios or mean difference with 95% confidence intervals. AF: atrial fibrillation; DBP: diastolic blood pressure; GTN: glyceryl trinitrate; mRS: modified Rankin Scale; SD: standard deviation; SSS: Scandinavian Stroke Scale; t-MMSE: telephone mini-mental state examination; TICS-M: modified telephone interview for cognition scale.

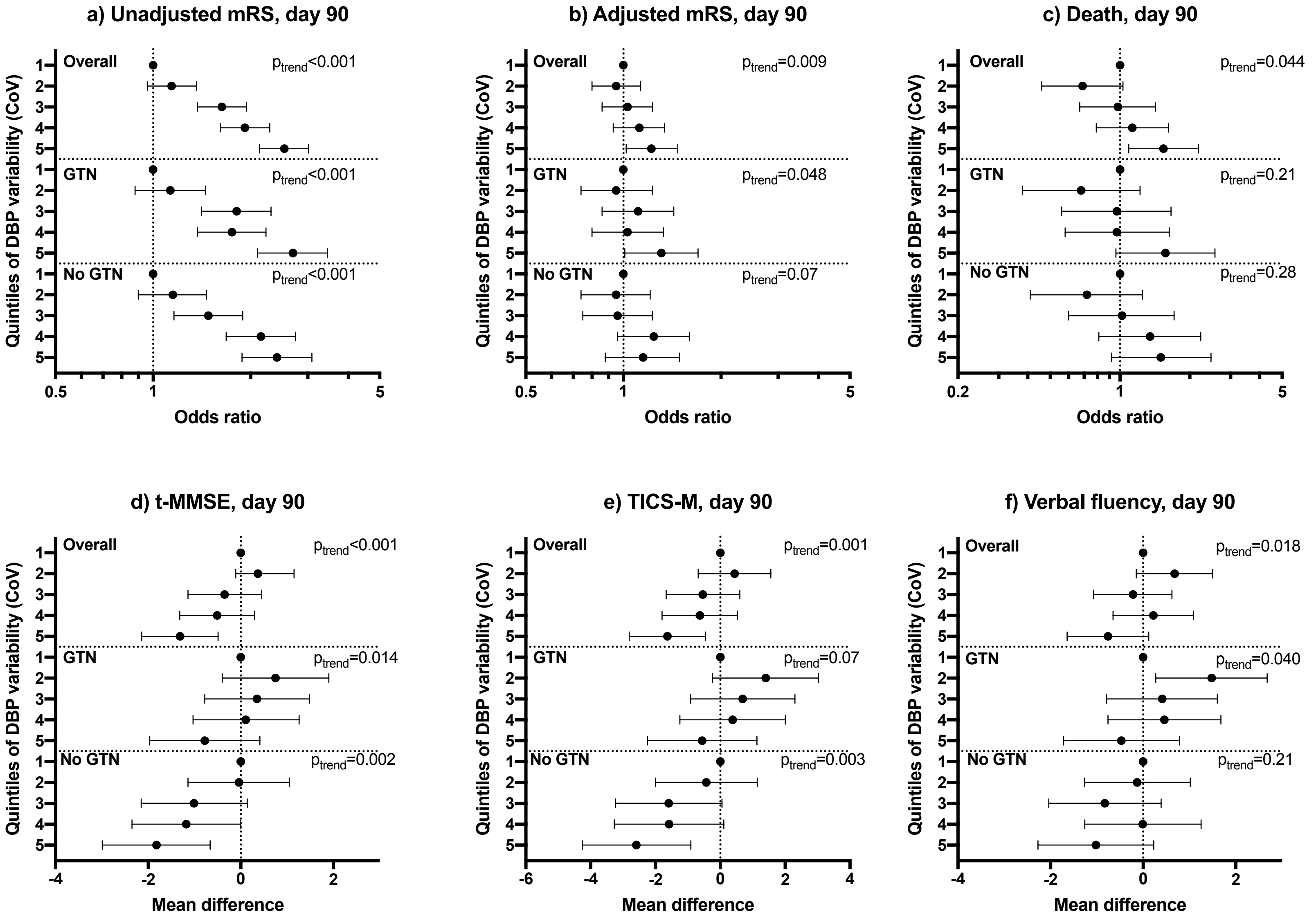
a) Unadjusted mRS, day 90
b) Adjusted mRS, day 90
c) Death, day 90
d) t-MMSE, day 90
e) TICS-M, day 90
f) Verbal fluency, day 90
Quintiles of DBP variability (CoV)
Overall
GTN
No GTN
ptrend<0.001
ptrend=0.009
ptrend=0.048
ptrend=0.07
ptrend=0.044
ptrend=0.21
ptrend=0.28
ptrend=0.014
ptrend=0.002
ptrend=0.001
ptrend=0.003
ptrend=0.018
ptrend=0.040
Odds ratio
Mean difference

Supplementary Figure III: Effect of DBP variability over days 1 to 7 (CoV) on day 90 outcomes

a-b: Quintiles of DBP variability over days 1 to 7 (CoV) vs. mRS at day 90 overall and in GTN and no GTN groups, with the first quintile as reference, (a) overall unadjusted (n=3978), (b) overall adjusted (n=3978).

c: Quintiles of DBP variability over days 1 to 7 (CoV) vs. death at day 90 overall (n=3982) and in GTN and no GTN groups, with the first quintile as reference.

d-f: Quintiles of DBP variability over days 1 to 7 (CoV) vs. cognitive outcomes at day 90 overall and in GTN and no GTN groups, with the first quintile as reference, (d) t-MMSE (n=2019), (e) TICS-M (n=2001), (f) verbal fluency (n=2352).

Ordinal (a-b) or binary (c) logistic or multiple linear (d-f) regression with adjustment for age, sex, baseline mRS, history of previous stroke, history of diabetes, final diagnosis, prior nitrate use, baseline SSS, total anterior circulation stroke, thrombolysis, feeding status, time to randomisation, baseline SBP, GTN vs no GTN, continue/stop. Data are odds ratios with 95% confidence intervals. AF: atrial fibrillation; CoV: coefficient of variation; DBP: diastolic blood pressure; GTN: glyceryl trinitrate; mRS: modified Rankin Scale; SSS: Scandinavian Stroke Scale; t-MMSE: telephone mini-mental state examination; TICS-M: modified telephone interview for cognition scale.

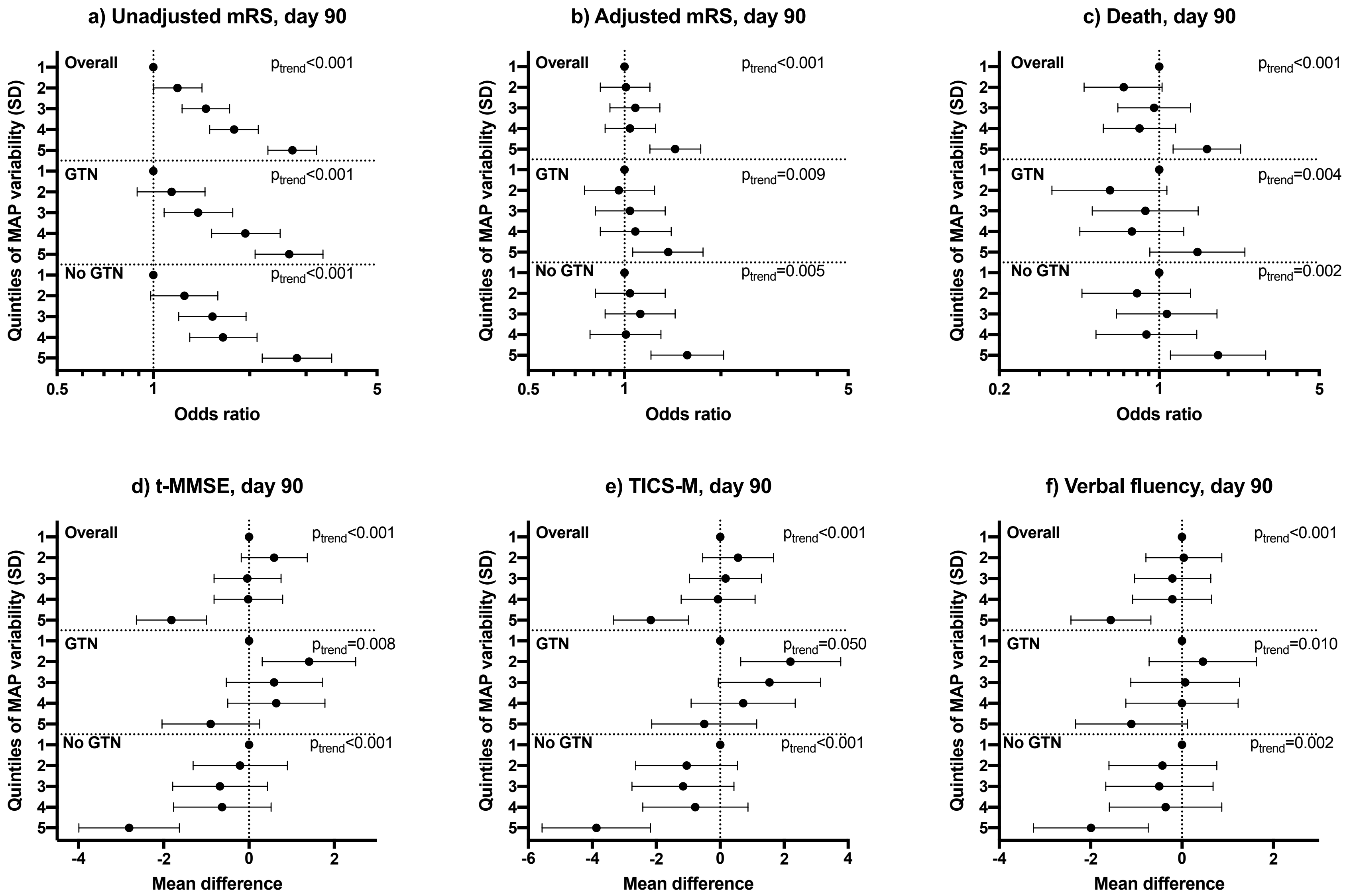
a) Unadjusted mRS, day 90
b) Adjusted mRS, day 90
c) Death, day 90
d) t-MMSE, day 90
e) TICS-M, day 90
f) Verbal fluency, day 90
Quintiles of MAP variability (SD)
Overall
GTN
No GTN
1
2
3
4
5
a) Overall p trend<0.001; GTN p trend<0.001; No GTN p trend<0.001
b) Overall p trend<0.001; GTN p trend=0.009; No GTN p trend=0.005
c) Overall p trend<0.001; GTN p trend=0.004; No GTN p trend=0.002
d) Overall p trend<0.001; GTN p trend=0.008; No GTN p trend<0.001
e) Overall p trend<0.001; GTN p trend=0.050; No GTN p trend<0.001
f) Overall p trend<0.001; GTN p trend=0.010; No GTN p trend=0.002
Odds ratio
0.5
1
5
0.2
Mean difference
-6
-4
-2
0
2
4

Supplementary Figure IV: Effect of MAP variability over days 1 to 7 (SD) on day 90 outcomes
a-b: Quintiles of MAP variability over days 1 to 7 (SD) vs. mRS at day 90 overall and in GTN and no GTN groups, with the first quintile as reference, (a) overall unadjusted (n=3978), (b) overall adjusted (n=3978).
c: Quintiles of MAP variability over days 1 to 7 (SD) vs. death at day 90 overall (n=3982) and in GTN and no GTN groups, with the first quintile as reference.
d-f: Quintiles of MAP variability over days 1 to 7 (SD) vs. cognitive outcomes at day 90 overall and in GTN and no GTN groups, with the first quintile as reference, (a) t-MMSE (n=2019), (b) TICS-M (n=2001), (c) verbal fluency (n=2352).
Ordinal (a-b) or binary (c) logistic or multiple linear (d-f) regression with adjustment for age, sex, baseline mRS, history of previous stroke, history of diabetes, final diagnosis, prior nitrate use, baseline SSS, total anterior circulation stroke, thrombolysis, feeding status, time to randomisation, baseline SBP, GTN vs no GTN, continue/stop. Data are odds ratios with 95% confidence intervals. AF: atrial fibrillation; GTN: glyceryl trinitrate; MAP: mean arterial pressure; mRS: modified Rankin Scale; SD: standard deviation; SSS: Scandinavian Stroke Scale; t-MMSE: telephone mini-mental state examination; TICS-M: modified telephone interview for cognition scale.

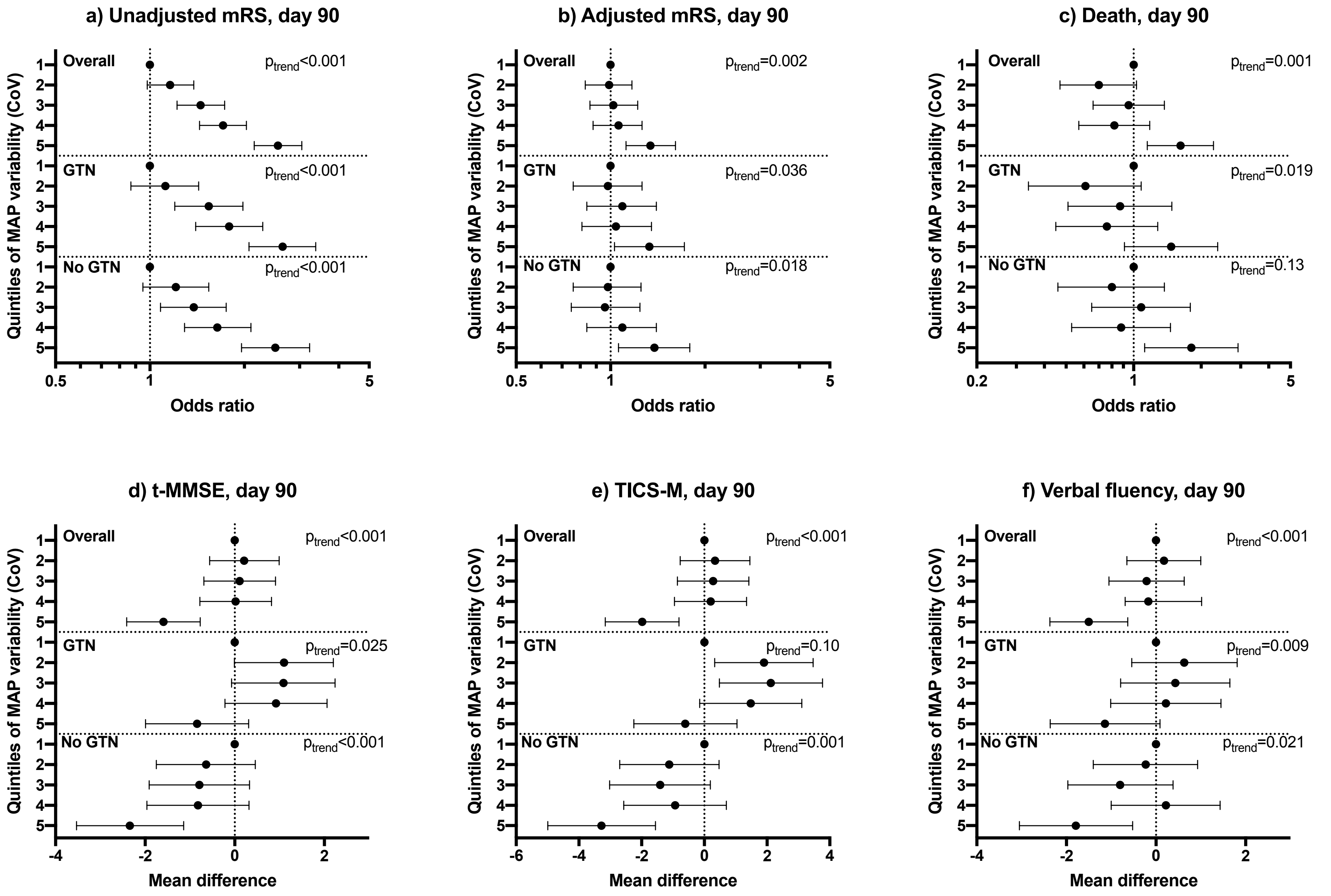
a) Unadjusted mRS, day 90
b) Adjusted mRS, day 90
c) Death, day 90
d) t-MMSE, day 90
e) TICS-M, day 90
f) Verbal fluency, day 90
Quintiles of MAP variability (CoV)
Overall
GTN
No GTN
ptrend<0.001
ptrend<0.001
ptrend<0.001
ptrend=0.002
ptrend=0.036
ptrend=0.018
ptrend=0.001
ptrend=0.019
ptrend=0.13
ptrend<0.001
ptrend=0.025
ptrend<0.001
ptrend<0.001
ptrend=0.10
ptrend=0.001
ptrend<0.001
ptrend=0.009
ptrend=0.021
Odds ratio
Mean difference

Supplementary Figure V: Effect of MAP variability over days 1 to 7 (CoV) on day 90 outcomes
a-b: Quintiles of MAP variability over days 1 to 7 (CoV) vs. mRS at day 90 overall and in GTN and no GTN groups, with the first quintile as reference, (a) overall unadjusted (n=3978), (b) overall adjusted (n=3978).
c: Quintiles of MAP variability over days 1 to 7 (CoV) vs. death at day 90 overall (n=3982) and in GTN and no GTN groups, with the first quintile as reference.
d-f: Quintiles of MAP variability over days 1 to 7 (CoV) vs. cognitive outcomes at day 90 overall and in GTN and no GTN groups, with the first quintile as reference, (a) t-MMSE (n=2019), (b) TICS-M (n=2001), (c) verbal fluency (n=2352).
Ordinal (a-b) or binary (c) logistic or multiple linear (d-f) regression with adjustment for age, sex, baseline mRS, history of previous stroke, history of diabetes, final diagnosis, prior nitrate use, baseline SSS, total anterior circulation stroke, thrombolysis, feeding status, time to randomisation, baseline SBP, GTN vs no GTN, continue/stop. Data are odds ratios with 95% confidence intervals. AF: atrial fibrillation; CoV: coefficient of variation; GTN: glyceryl trinitrate; MAP: mean arterial pressure; mRS: modified Rankin Scale; SSS: Scandinavian Stroke Scale; t-MMSE: telephone mini-mental state examination; TICS-M: modified telephone interview for cognition scale.
